# Correction: Preparation and characterization of a novel drug-loaded Bi-layer scaffold for cartilage regeneration

**DOI:** 10.1039/d2ra90060b

**Published:** 2022-06-14

**Authors:** Yunqing Yue, Peihu Xu, Zhixin Lei, Kebi Li, Jingyi Xu, Jing Wen, Sining Wang, Wanting Cheng, Sihui Lin, Zhijun Huang, Haixing Xu

**Affiliations:** Department of Pharmaceutical Engineering, School of Chemistry, Chemical Engineering and Life Science, Wuhan University of Technology 430070 China Whutxph68@126.com xhx040328@whut.edu.cn

## Abstract

Correction for ‘Preparation and characterization of a novel drug-loaded Bi-layer scaffold for cartilage regeneration’ by Yunqing Yue *et al.*, *RSC Adv.*, 2022, **12**, 9524–9533, https://doi.org/10.1039/D2RA00311B.

The authors regret that part of Fig. 1 and the Graphical Abstract were reproduced from a published article without appropriate acknowledgment. The reference to be included is Girão *et al.*, *Compos. B. Eng.*, 2018, **154**, 99–107, https://doi.org/10.1016/j.compositesb.2018.08.001.

Girão *et al.* have granted permission for part of their Fig. 1 to be used in “Preparation and characterization of a novel drug-loaded Bi-layer scaffold for cartilage regeneration”.

The Royal Society of Chemistry apologises for these errors and any consequent inconvenience to authors and readers.

## Supplementary Material

